# Dynamics and stability of polymorphic human telomeric G-quadruplex under tension

**DOI:** 10.1093/nar/gku581

**Published:** 2014-07-10

**Authors:** Huijuan You, Xiangjun Zeng, Yue Xu, Ci Ji Lim, Artem K. Efremov, Anh Tuân Phan, Jie Yan

**Affiliations:** 1 Mechanobiology Institute, National University of Singapore, 5A Engineering Drive 1, 117411, Singapore; 2 School of Physical and Mathematical Sciences, Nanyang Technological University, 21 Nanyang Link, 637371, Singapore; 3 Graduate School for Integrative Sciences and Engineering, National University of Singapore, 28 Medical Drive, 117456, Singapore; 4 Centre for Bioimaging Sciences, National University of Singapore, 14 Science Drive 4, 117546, Singapore; 5 Department of Physics, National University of Singapore, 2 Science Drive 3, 117542, Singapore

## Abstract

As critical DNA structures capping the human chromosome ends, the stability and structural polymorphism of human telomeric G-quadruplex (G4) have drawn increasing attention in recent years. This work characterizes the equilibrium transitions of single-molecule telomeric G4 at physiological K^+^ concentration. We report three folded states of telomeric G4 with markedly different lifetime and mechanical stability. Our results show that the kinetically favored folding pathway is through a short-lived intermediate state to a longer-lived state. By examining the force dependence of transition rates, the force-dependent transition free energy landscape for this pathway is determined. In addition, an ultra-long-lived form of telomeric G4 structure with a much stronger mechanical stability is identified.

## INTRODUCTION

In addition to the canonical double helix B-form structure, DNA can adopt other structures, such as the duplex structures Z-DNA, S-DNA, as well as triplex and tetraplex, etc. (1,2). The tetraplex structures formed on G-rich sequences, referred to as G-quadruplex (G4), have drawn extensive attention recently. Increasing evidences have suggested that such structures are ubiquitous in the genome and may play various crucial regulatory functions such as regulation of chromosome stability and gene transcriptions (3,4). More than 300 000 putative quadruplex sequences have been found in the human genome (5,6) and many of them are located within the biologically functional regions such as telomeres, the ribosomal DNA, the immunoglobulin switch region (7) and promoters (8).

The human telomeric G4 forms in a specialized region of eukaryotic chromosomes, called telomeres, at the ends of the chromosomes. Human telomeric DNA consists of several kilobases of double-stranded DNA (dsDNA) tandem repeats of 5′-TTAGGG sequence, ended with a terminal single-stranded 3′ overhang of 100–200 nucleotides (9). The terminal single-stranded DNA (ssDNA) has the potential to fold into G4 structures which caps the chromosomal ends (1). Besides protection of the 3′ overhang end of chromosome, telomeric G4 plays a critical role in inhibiting the activity of telomerase (10). Telomerase is a telomere binding enzyme, which elongates and regulates the length of telomeres (11). The telomerase activity has been reported to be much higher in cancer cells than in normal cells (12). Small molecules stabilizing telomeric G4 have been shown to effectively inhibit telomerase activity and has an anti-cancer activity (13). Thus, the stability of telomeric G4 is also crucial for anti-cancer drug developments.

Telomeric G4 is made of multiple stacked planes each consisting of four coplanar guanines linked through Hoogsteen base pairing with a centrally located monovalent cation, K^+^ or Na^+^ (14,15). The structures of telomeric G4 are highly polymorphic: different structures have been reported, including the parallel G4 with four parallel strands (14), the anti-parallel G4 with two diagonal anti-parallel strands (15) and hybrid G4 with mixed parallel and anti-parallel strands (16–18). The formation of G4 structures is strongly dependent on the type of monovalent cations. Because of the significantly higher intracellular concentration of K^+^ (typically ∼100 mM) than Na^+^ (typically ∼10 mM), K^+^ induced G4 structures are considered as the predominant structures under physiological conditions.

Many studies have been directed to measure the stability of K^+^ induced telomeric G4 using a variety of experimental approaches including circular dichroism (CD), UV absorption spectrum and fluorescence resonance energy transfer (FRET) (19–22). In such studies, the melting curve of telomeric G4 has been measured by changing temperature or K^+^ concentration. The free energy cost of unfolding of K^+^ induced telomeric G4, Δ*G*_0_, has been reported in a wide range of 3.4–14.8 kcal mol^−1^ (20,23,24). Consistent with telomeric G4 as a stable structure, a fast folding rate of 16–50 s^−1^ and a slow unfolding rate of 1.3 × 10^−3^ s^−1^ have been reported by quick changing K^+^ concentration (25,26).

As multiple structures may co-exist in the K^+^ solutions, more recently efforts have been directed to investigate dynamics of single telomeric G4 molecules in order to detect different species including short lifetime transitions. Multiple folded conformations have been reported by tracking the dynamics of the single-molecule FRET (smFRET) efficiency of a single tethered DNA containing the telomeric repeats (22). However, in that study destabilizing low K^+^ concentrations (<10 mM) were used to increase the transition rates in order to obtain sufficient number of transitions for statistical analysis. In physiological level of ∼100 mM K^+^, due to slow unfolding rates, smFRET has been applied to study the populations of folded G4 structures instead of transition dynamics, which have revealed existence of multiple folded conformations (22,27). The studies of the dynamics for telomeric G4 have not been carried out in physiological concentration range of K^+^ (∼100 mM).

The slow unfolding rate of telomeric G4 structures in physiological K^+^ concentration can be overcome by applying forces to the G4 molecules. The so-called Bell's model (28) describes the force-dependent unfolding rate as:

(1) \begin{equation*} k_{\rm u}(f)=k_{\rm u}^0\exp (x_{\rm u}f/k_{\rm B}T), \end{equation*}

where $k_{\rm u}^0$ is the unfolding rate at zero force, *x*_u_ is the distance from the native state to the transition state, *k*_B_ is Boltzmann constant and *T* is the absolute temperature. The Bell's model shows that unfolding rate exponentially increases with force.

When the molecule is stretched with force linearly increasing with time at a loading rate of *r*, an unfolding force distribution, *p*(*f*), can be derived from the Bell's model:

(2) \begin{equation*} p(f)= \frac{k_{\rm u}^0}{r} \exp \lbrace \frac{x_{\rm {\rm u}}f}{k_{\rm B}T}+\frac{k_{\rm B} T k_{\rm u}^0}{x_{\rm u}r}[1-\exp (\frac{x_{\rm u}f}{k_{\rm B}T})]\rbrace , \end{equation*}

which predicts a single force peak located at $f=(k_{\rm B}T/x_{\rm u})\ln (x_{\rm u}r/k_{\rm B} T k_{\rm u}^0)$ (29).

In previous single-molecule stretching experiments, telomeric G4 unfolding in 100 mM K^+^ has been achieved by stretching the molecule at certain loading rates, resulting in quick telomeric G4 unfolding typically at forces ∼21 pN at a loading rate of 5.5 pN/s (30). Folding of unfolded telomeric G4 was achieved by dropping force to a much lower value. Because unfolding and folding transitions do not occur at the same force, such experimental procedure does not determine Δ*G*_0_ directly. Instead, a non-equilibrium approach based on the Jarzynski's theorem (31) has been used to estimate Δ*G*_0_ to be around 9.8 ± 0.4 kcal mol^−1^ (30), which is close to the higher boundary reported from previous melting curve measurements. As applying Jarzynski's theorem requires a large number of stretch-relax procedures on the same G4 tether and an assumption that work involved in each stretch-relax procedure is close to *k*_B_*T*, Δ*G*_0_ obtained by this method still needs to be confirmed by more direct equilibrium measurements.

In this work, we directly observed the equilibrium transition of single-molecule telomeric G4 under constant forces at physiological relevant K^+^ concentration of 100 mM, using ultra-stable magnetic tweezers (32). From the long time trace that contains numerous folding and unfolding transitions at constant forces, multiple folded structures formed on telomeric G4 sequence have been identified and characterized by their lifetimes and mechanical stability.

## MATERIALS AND METHODS

### DNA construct and NMR

All DNA oligonucleotides were purchased form Integrated DNA Technologies (IDT). The 5′-thiol labeled 1449 bp and 5′-biotin labeled 601 bp dsDNA handles were prepared by PCR using DreamTaq DNA polymerase (Thermo Scientific) on lambda phage DNA template (New England Biolabs, NEB) using 5′-thiol and 5′-biotin primers. Both dsDNA handles have high GC content (>60%) to prevent DNA melting when DNA is held at high forces or during DNA overstretching transition (33–36). PCR products were purified using PureLink PCR purification kit (Invitrogen) and digested with BstXI restriction enzyme (NEB). Telomeric G-rich oligo, dsDNA handles were ligated using T4 DNA ligase (NEB). The ligated product (2042 bp with 26 nt) was purified by gel extraction with PureLink kit (Invitrogen). More details of the DNA constructs are included in SI: ‘DNA oligonucleotides’ and SI: Figure S1. 1D nuclear magnetic resonance (NMR) experiment was conducted on a Bruker AVANCE 600 MHz spectrometer at 25°C. JR-type pulse sequence was used for water signal suppression.

### Single DNA stretching experiments

A flow chamber of 10–20 μl in volume was constructed on a (3-Aminopropyl)triethoxy silane (APTES) (Sigma-Aldrich) functionalized coverslip (32 × 24 mm). Thiol-end of DNA was covalently attached to the amine group of APTES via sulfo-SMCC crosslinker (Thermo Scientific). The APTES coverslip was first reacted with 1 mg/ml sulfo-SMCC (dissolved in 1× phosphate buffered saline (PBS) pH 7.4 buffer) for 30 min. After washing out unbound sulfo-SMCC, thiol-labeled G4 DNA construct was introduced into the chamber and incubated for 30 min. The chamber was then blocked with BSA solution (10 mg/ml BSA, 1mM 2-mercaptoethanol, 1 × PBS pH 7.4 buffer) for more than 2 h before experiments. After DNA constructs were bound to the surface, 2.8 μm-diameter streptavidin-coated paramagnetic beads (Dynal M-280, Life technologies) were introduced to attach to the biotin end of DNA. Finally, the buffer was changed to assay buffer (10 mM Tris-HCl, pH 8.0, 100 mM KCl) for single-molecule stretching experiments.

**Figure 1.**
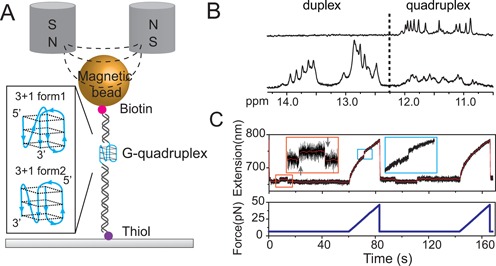
Experimental setup and procedure. (**A**) Schematic diagram of G4 DNA and magnetic tweezers. The 26 mer ssDNA of four-repeat human telomeric sequence d(TTGGG(TTAGGG)_3_TTT) is sandwiched bewteen a lower 1449 bp and an upper 601 bp dsDNA handles. The DNA construct is tethered between a 2.8 μm-diameter paramagnetic bead via biotin-streptavidin linkage and an amine functionalized coverslip surface through covalent cross-linker sulfo-SMCC. Inset shows two possible hybrid-type G4 structures that may form on the sequence (16,17). (**B**) Imino proton NMR spectrum of telomeric G4 sequence d(TTGGG(TTAGGG)_3_TTT) (top) and the 1:1:1 mixture of three sequences, d(CGAGTCTGTGCACAAGGTGC), d(CTACTGACCTGGCTGC) and d(CTTGTGCACAGACTCGTTGGG(TTAGGG)_3_TTTGCAGCCAGGTCAGTAGCGAC) (bottom). The mixture is expected to form a G-quadruplex in the centre flanked by two 16-bp Watson–Crick duplexes at the 5′- and 3′-ends respectively (underlined sequences). (**C**) Typical force responses of G4 DNA in two repeating stretching cycles (original and smoothed extension data are shown in black and red, respectively). In each cycle, a constant force was maintained at 6.5 pN for 60 s then increased to 50 pN at a constant loading rate of 2 pN/s. In the first cycle, at the constant force of 6.5 pN, two extension states with an extension difference of ∼6 nm were observed, indicated by an unfolding transition (up-arrow) followed by a refolding transition (down-arrow) in the zoom-in inset in the orange rectangle. During the subsequent force-increase scan at 2 pN/s, a typical G4 unfolding indicated by a sudden extension jump with a step size of ∼8 nm occurred at ∼25 pN (marked in cyan rectangle). In the second cycle after force was jumped back to 6.5 pN, a refolding transition and a following unfolding transition were observed. In the subsequent force-increase scan at 2 pN/s, G4 unfolding was not observed.

Ultra stable vertical magnetic tweezers built in lab were used to stretch the DNA constructs using a pair of magnets on the top of the chamber (Figure 1A). The magnetic tweezers were controlled by in-house-written LabVIEW program (National Instruments). The extension change of the construct was recorded with a sampling rate of ∼200 frames per second. Force was controlled by changing the distance between the permanent magnets and flow chamber. Loading rate control was achieved by moving the magnets through a programmed trajectory. The magnetic tweezers have a spatial resolution for bead stuck on surface of ∼2 nm, and the force calibration has a relative error of <10% (32). More details of the magnetic tweezers design, the force and loading rate control, as well as the force calibration were detailed in our previous publications (32,37). All experiments were carried out at our lab room temperature of 21−23 °C.

## RESULTS

### Folding and unfolding telomeric G4 at constant forces

In our experiments, a single-stranded telomeric G4 DNA formed on 5′TTGGG(TTAGGG)_3_TTT sandwiched between two dsDNA handles was stretched using ultra-stable magnetic tweezers (Figure 1A) (37). In each experiment, a single-tether attachment was ensured by the highly specific B-to-S transition of the dsDNA handles during DNA overstretching characterized by ∼1.68-fold extension increase in a narrow force range of ∼65 pN (33–36).

The sequence used in our experiment was confirmed to form G4 by NMR experiment (Figure 1B, top panel). To confirm the G4 formation in the context of single-molecule mechanical experiments, i.e. when this sequence is embedded between duplex handles, we performed NMR experiments on a construct containing short Watson–Crick duplexes at the 5′- and 3′-ends of the same G-rich fragment. Indeed, the NMR spectrum of this construct (Figure 1B, bottom panel) showed imino proton signals from 10.5 to 14 ppm, corresponding to the formation of both G-quadruplex (10.5–12 ppm) and Watson–Crick duplexes (12–14 ppm).

Figure 1C shows the force responses of the telomeric G4 formed in our DNA construct in two representative repeating stretching cycles. In each cycle, the tether was held at a low constant force of 6.5 ± 0.6 pN for 1 min followed by stretching at a constant loading rate of 2 pN/s up to ∼50 pN. During 1 min of holding the DNA tether at ∼6.5 pN, stepwise extension changes with a step size of ∼6 nm were observed (Figure 1B, inset). In the subsequent constant loading rate stretching, two types of force responses were observed: (i) If the DNA was in the lower extension state prior to the constant loading rate stretching, an abrupt unfolding transition with a step size of ∼8 nm was observed at a force of ∼20 pN (Figure 1C, first stretching cycle). (ii) If the DNA was in the longer extension state prior to the constant loading rate stretching, such unfolding step was not observed (Figure 1C, second stretching cycle). These results indicate that the stepwise extension changes at ∼6.5 pN are spontaneous telomeric G4 folding and unfolding transitions, and suggest that it is possible to observe equilibrium transitions of structures formed in the telomeric G4 sequence at low forces.

The unfolding force distribution *p*(*f*) obtained from constant loading rate stretching can be fitted with *p*(*f*) predicted by Equation (2) with *x*_u_ = 0.8 ± 0.2 nm and $k_{\rm u}^0$ = 0.009 ± 0.008 *s*^−1^ (average ± standard deviation) (SI: Figure S2). A peak unfolding force of 17 ± 5 pN (average ± standard deviation) agrees with the value of 21 ± 1 pN reported in previous works conducted at a similar loading rate (5.5 pN/s) (30,38). The histogram of the unfolding step sizes can be fitted with Gaussian distribution with a peak value of 8 ± 2 nm (average ± standard deviation) at the force range (Figure S2), which is consistent with unfolding of 21 nucleotides based on the known force response of ssDNA and the size of G4 (39) (SI: ‘Force-extension curve for ssDNA’).

### Dynamics of folding and unfolding transitions reveal three folded telomeric G4 conformations

**Figure 2.**
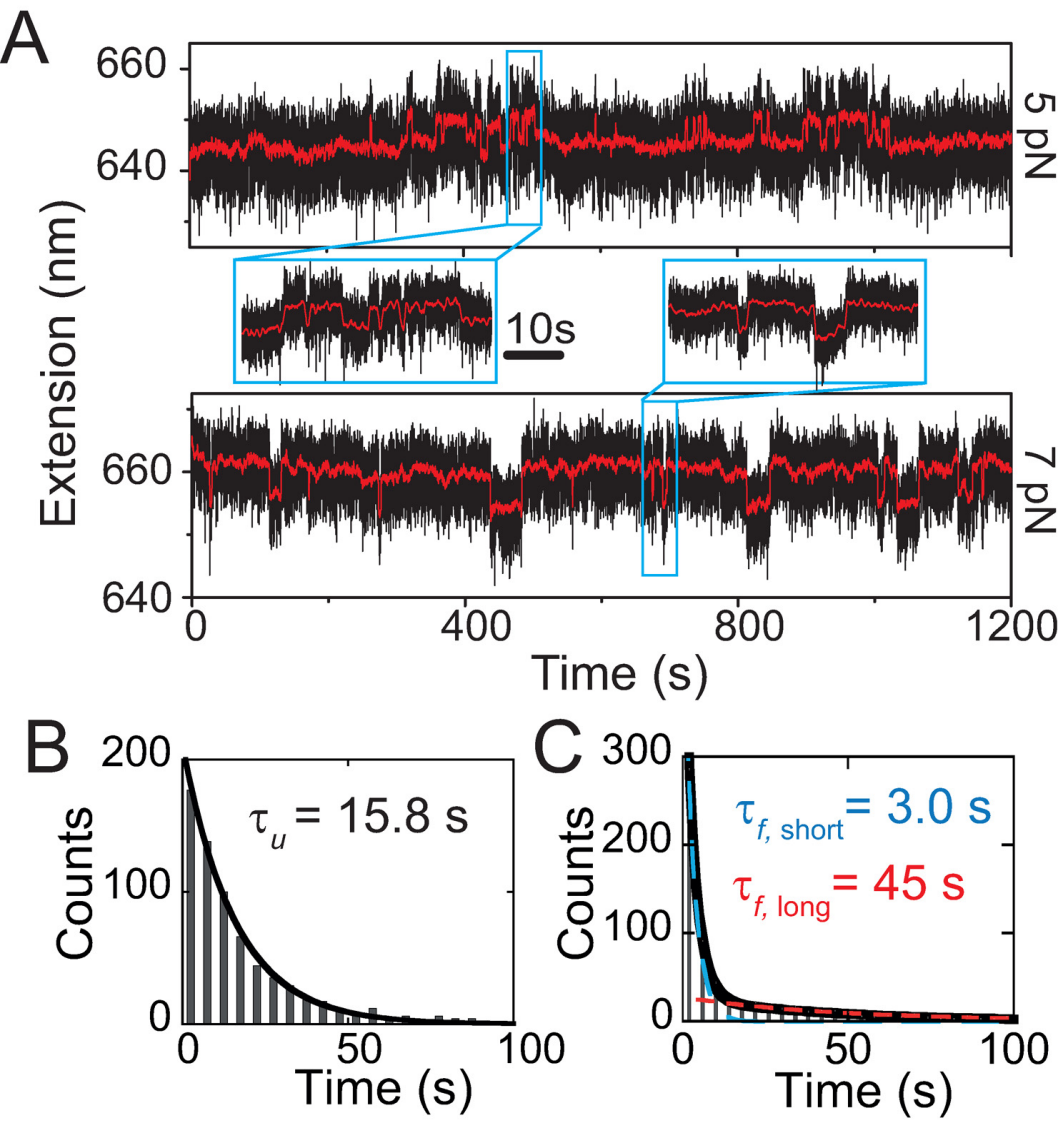
Equilibrium folding and unfolding dynamics under constant forces. (**A**) Representative time traces (black dots) of the extension fluctuation of a single telomeric G4 tether recorded at 5.0 ± 0.5 pN (upper panel) and 7.0 ± 0.7 pN (lower panel). The red lines show smoothed time traces by adjacent average of data using a 0.5 s sliding time window. Insets show zoom-in time traces containing rapid folding and unfolding transitions. (**B**) Histogram of the dwell time of the longer extension state (i.e. unfolded G4) recorded at 5.0 ± 0.5 pN, which is fitted by a single-exponential decay function with time constant of *τ*_u_ = 15.8 ± 0.4 s (black curve, average ± standard error). (**C**) Histogram of the dwell time of the shorter extension state (i.e. folded G4) recorded at the same force, which cannot be fitted by a single-exponential decay function. Therefore, a double-exponential decay function with two time constants of *τ*_*f*, short_ = 3.0 ± 0.1 s and *τ*_*f*, short_ = 45 ± 4 s was used for the fitting (black curve), which is the sum of two individual single-exponential fitting using the respective time constants. Data in panels (B) and (C) include 683 folding events and 684 unfolding events collected from 13 independent telomeric G4 DNA tethers.

The dynamics of telomeric G4 folding and unfolding at constant forces was investigated. Two representative time traces of the extension fluctuation of a telomeric G4 DNA construct at two different forces (5 and 7 pN) are shown in Figure 2A, where G4 folding and unfolding transitions are indicated by sudden stepwise extension changes detected by a noise beating algorithm (40). At 5 pN, the folded state (the shorter extension state) is predominant; while at 7 pN, the unfolded state (the longer extension state) becomes predominant.

The dwell time histograms of the unfolded state obtained at each force can be fitted with a single exponential decay function $A\text{exp}(-k_{{\rm u}{\rightarrow }f}t)$, where *k*_u → *f*_ indicates the folding rate constant and the reciprocal *τ*_u_ = 1/*k*_u → *f*_ is the lifetime of the unfolded state. *τ*_u_ at different forces are fitted to be 15.8 ± 0.4 s (5 pN), 53 ± 5 s (6 pN) and 101 ± 11 s (7 pN), respectively (Figure 2B and SI: Figures S3 and S4). Here the errors are fitting standard errors. At each force, data recorded from at least nine independent tethers are combined together to obtain the final fitting.

In contrast to the folding transition, the dwell time histograms of the folded states cannot be fitted with single exponential function while a double-exponential function $A_1\text{exp}(-k_{f{\rightarrow }{\rm u,short}}t)+ A_2\text{exp}(-k_{f{\rightarrow }{\rm u,long}}t)$ with two rate constants *k*_*f* → u, short_ and *k*_*f* → u, long_ can. Two distinct lifetimes of the folded states are determined to be (*τ*_*f*, short_, *τ*_*f*, long_) = (3.0 ± 0.1, 45 ± 4 s) at 5 pN (Figure 2C). At 6 and 7 pN, they are fitted to be (2.1 ± 0.2, 25.0 ± 6.3 s) and (3.2 ± 0.5, 16.6 ± 4.9 s), respectively (SI: Figure S4). Hereafter the folded state characterized by the lifetime of several seconds is referred to as the short-lived state, and the one characterized by a lifetime of tens of seconds is referred to as the long-lived state. The zoom-in time traces in Figure 2A show rapid unfolding and folding transitions of the short-lived state.

**Figure 3.**
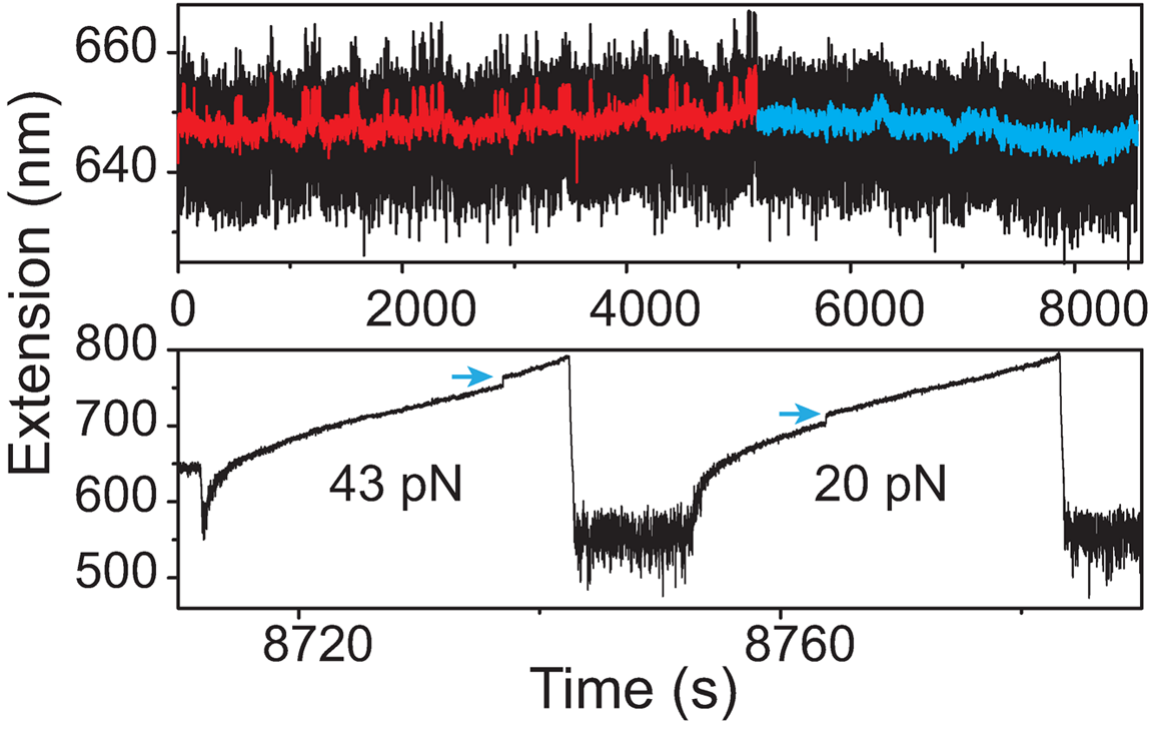
Ultra long-lived G4 state. Upper panel shows a long time trace of a telomeric G4 DNA extension fluctuation at ∼5 pN (original and smoothed data are shown in black and red, respectively). In the first 5100 s, stepwise fluctuation with similar kinetics to Figure 2A was observed (red smoothed data for clarity). After 5100 s, the tether extension remained at the folded extension state for 1 h (cyan smoothed data for clarity). Lower panel shows the extension change of the ultra-long lived telomeric G4 being stretched at 2 pN/s, where unfolding occurred at ∼43 pN (the first stretch). After subsequent refolding at 1 pN for 10 s, unfolding occurred at a much lower force of ∼20 pN.

Besides the short- and long-lived states, a third folded state characterized by an ultra-long lifetime was identified. In the long time trace recorded at ∼5 pN (Figure 3, upper panel), stepwise extension fluctuations similar to those in Figure 2A were observed in the first 5100 s (red smoothed data for clarity). However, after 5100 s, the stepwise fluctuations disappeared and the G4 DNA remained folded for 1 h (blue smoothed data for clarity). The ultra long lifetime of this folded state indicates that it is different from the long-lived state discussed in the previous paragraph. Hereafter this folded state is referred to as the ultra-long-lived state.

This state has a remarkable mechanical stability, unfolding at 43 pN (Figure 3, lower panel, the first stretch) during a subsequent constant loading rate stretching of 2 pN/s, which is significantly higher than the peak unfolding force of 17 ± 4 pN revealed from the histogram in Figure 1C. After this ultra long-lived structure was unfolded, the tether was refolded at 1 pN for 20 s. In the next constant loading rate stretch it was unfolded at ∼20 pN. Similar long time measurements (1–4 h) were repeated for 15 times and such ultra-long-lived state was observed for 6 times, which remained folded in 5–7 pN for at least 1000 s. Unfolding these six ultra long-lived structures at 2 pN/s revealed unfolding forces in the range of 42 ± 5 pN (average ± standard deviation). Together with results revealed in Figures 2 and 3, we conclude that at least three folded states characterized by different lifetimes and mechanical stability exist.

### G4 folding to the long-lived state is through the short-lived state as an intermediate

The folding kinetics can also be obtained by examining the time evolution of folding probability *p*_fold_(*t*), which was obtained by the following procedure. Firstly, a folded telomeric G4 was unfolded by constant loading rate stretching at 2 pN/s, followed by jumping to a lower force *f* for refolding by holding the tether for certain time *t* at the force. Successful refolding was indicated by an unfolding event in a subsequent constant loading rate stretching. Repeating such procedure *N* times, the *p*_fold_(*t*) was obtained by *M*/*N*, where *M* is the number of successful folding (SI: Figure S5). At each force *f* and holding time *t*, such stretching-folding procedure was repeated for more than 100 times from more than four independent tethers to obtain *p*_fold_(*t*). Within our holding time scale of <120 s, the formation of the ultra long-lived state is negligible (SI: Figure S6).

**Figure 4.**
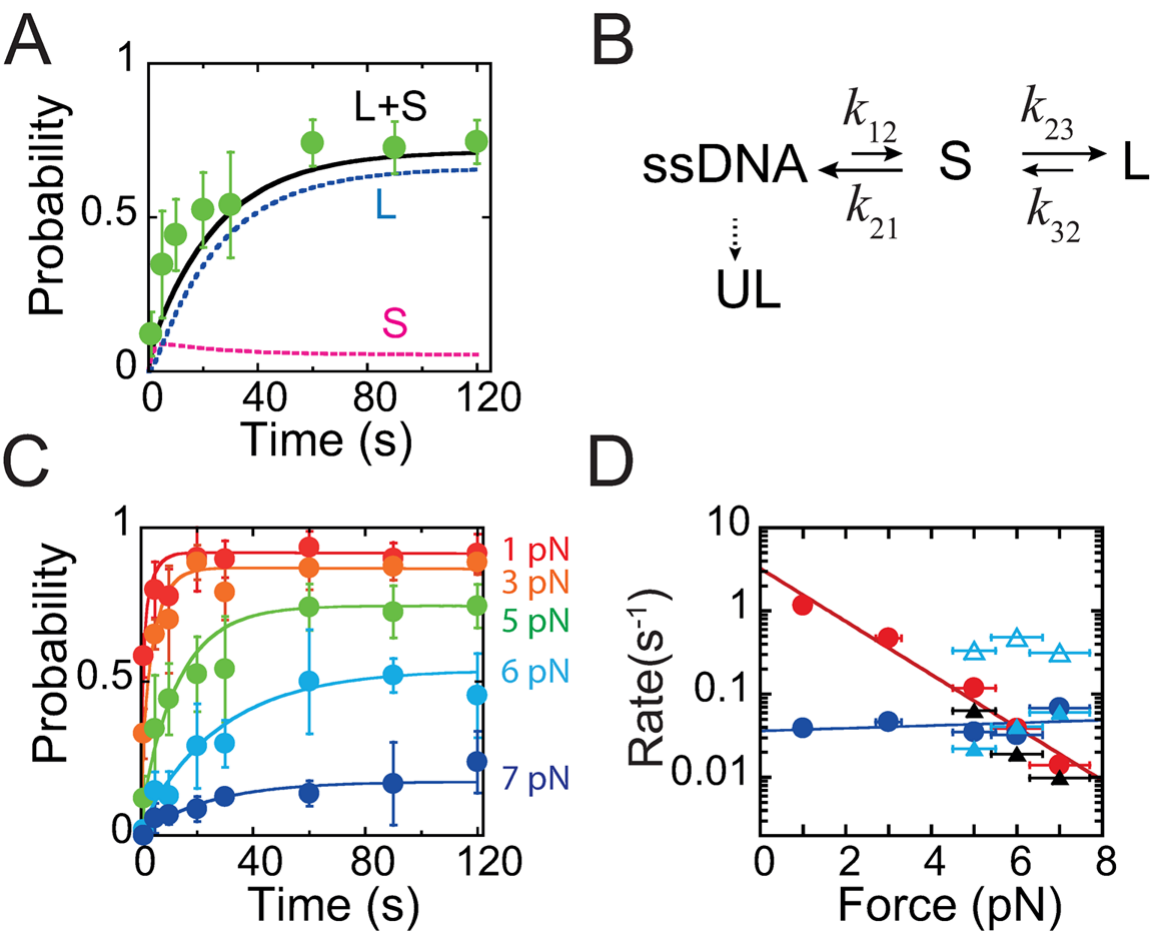
Telomeric G4 folding and unfolding kinetics and stability. (**A**) Green solid circles indicate experimental measured *p*_fold_(*t*) at 5 pN. Black curve is the predicted *p*_fold_(*t*) based on the sequential model in (**B**), which is the sum of the probabilities of the long-lived state (L, blue curve) and the short-lived state (S, purple curve). (B) Sketch of the sequential transition model, in which the long-lived state, short-lived state and ultra-long-lived state are indicated by ‘L’, ‘S’ and ‘UL’, respectively. (**C**) Filled circles of different colors indicate experimental *p*_fold_(*t*) measured at different forces. Each data point was calculated based on more than 100 refolding/unfolding cycles from at least four molecules. The solid lines with corresponding colors are best fitting based on the Master equation of the sequential model. (**D**) The fitted *k*_12_ (red solid circles) and *k*_32_ (blue solid circles) at different forces. The black triangles are *k*_u_ → *f* determined in dwell time analysis at constant forces of 5, 6 and 7 pN, respectively. The open and solid cyan triangles are *k*_*f* → u, short_ and *k*_*f* → u, long_ of the short-lived (open triangle) and long-lived (solid triangle) states, respectively, which are directly determined by dwell time analysis. X-axis error bars reflect a ∼10% error in force determination. Y-axis error bars are the standard deviation.

Figure 4A shows that *p*_fold_(*t*) obtained at 5 pN increases with time and nearly reaches a steady state at >60 s with a probability of ∼0.8. A sequential model where folding is through the short-lived state as an intermediate state to the long-lived state was considered to explain the result, plus a branched folding to the ultra long-lived state although it does not play a role in *p*_fold_(*t*) obtained within a time scale of 120 s. In this model, the transition rates can be directly determined by dwell time analysis at the same force as shown in Figure 2B-C: *k*_12_ = *k*_u → *f*_,*k*_21_ = *k*_*f* → u, short_, *k*_32_ = *k*_*f* → u, long_, *k*_23_ = *k*_21_*N*_long_/*N*_short_ (SI: Table S1). Here, *N*_long_ and *N*_short_ are the numbers of the long-lived and short-lived states counted from the dwell time histogram, which ratio equals to the ratio of transition rates of *k*_23_ and *k*_21_. With these model parameters, *p*_fold_(*t*) was solved from the Master equations using an in-house written Matlab code based on the Runge–Kutta method (SI: ‘Kinetic models’), which was then compared with the experimental data. Using the rates determined at 5 pN, the predicted *p*_fold_(*t*) from the sequential model (Figure 2A, solid line) reasonably agrees with the experimental data. Similar analysis at 6 and 7 pN also show good agreement between the experimental data and the predictions by the sequential model. An alternative parallel model with competitive parallel folding into different folded states was also considered; however, it does not fit the experimental data as good as the sequential model (SI: Figure S7).

### Force-dependent telomeric G4 folding and unfolding kinetics and stability

In order to obtain the force dependence of folding and unfolding rates in a broader force range, the theoretical predicted *p*_fold_(*t*) based on the sequential model and experimental data obtained at different forces were compared, treating the transition rates as fitting parameters. The five parameters in the sequential model can be reduced based on the knowledge of the rates that were directly measured in 5–7 pN. Due to the transient nature of the short-lived state, the outward transition rates from the short-lived state (*k*_21_ and *k*_23_) are much faster that the inward transition rates (*k*_12_ and *k*_32_). In 5–7 pN, *k*_21_ ∼*k*_23_ have a weak dependence on force and are about 10-folds faster than (*k*_12_ and *k*_32_). This makes the short-lived state quickly relaxed to a steady state in a few seconds (Figure 4A, pink curve).

Simply set *k*_23_ = *k*_21_ = *αk*_32_ (*α* ≥ 5), the parameter pair (*k*_12_, *k*_32_) were varied to search for the best fitting values. We found that the best fitting values of (*k*_12_, *k*_32_) are insensitive to change of *α* > 5 (SI: ‘Kinetic models’), and the values obtained at *α* = 10 are plotted in Figure 4D. The results show that the folding rate *k*_12_ (solid red circles) has a strong force dependence: increasing force from 1 to 7 pN, it exponentially decreases by more than 100-folds. In contrast, unfolding rate *k*_32_ is much less sensitive to force change.

The force-dependence of the transition rates contains information of the transition energy barrier, which can be understood by the Bell's model (Equation 1). The sensitivity of *k*_12_ and the insensitivity of *k*_32_ to force imply a large transition distance for folding to the short-lived state and a small transition distance for the unfolding from the long-lived stated to the short-lived state. Fitting to the Bell's model the zero force transition rates ($k_{12}^0$, $k_{32}^0$) and transition distances (${\Delta } x_{12}^{\ddagger }$, ${\Delta} x_{32}^{\ddagger }$) are respectively determined to be (1.6−6.7, 0.02−0.07 s^−1^ with 95% confidence) and (3.0 ± 0.7, 0.2 ± 0.6 nm) (average ± standard deviation), respectively.

In the force range 5–7 pN, the fitted *k*_12_(*f*) overlaps with the folding rate directly measured by dwell time analysis (solid black triangles), as shown in Figure 4D. As expected, the fitted *k*_32_(*f*) also overlaps with the unfolding rate of the long-lived state (solid cyan triangles) determined by the dwell time analysis. The unfolding rate of the short-lived state determined by dwell time analysis is also plotted for comparison (open cyan triangles).

The force dependent free energy cost for unfolding the short- and long-lived states to ssDNA, Δ*G*_ssDNA, short_(*f*) = *G*_ssDNA_(*f*) − *G*_short_(*f*) and Δ*G*_ssDNA, long_(*f*) = *G*_ssDNA_(*f*) − *G*_long_(*f*), are calculated from the transition rates directly obtained at three constant forces (5, 6 and 7 pN) from dwell time analysis by $\Delta G_{{\rm ssDNA}, {\rm short}}(f)=k_{\rm B}\text{T}\ln (k_{12}/k_{21})$ and $\Delta G_{{\rm ssDNA}, {\rm long}}(f)=k_B\text{T}\ln [k_{12}k_{23}/(k_{32}k_{21})]$, respectively.

Δ*G*(*f*) between ssDNA and a folded state contains a force-independent term that is determined by molecular interactions, Δ*G*_0_, and a force-dependent term that comes from the different conformational force responses between a folded G4 and the unfolded ssDNA, which can be analytically expressed as (41):

(3) \begin{equation*} \Delta G(f) = \Delta G_0 + \Delta \phi (f), \end{equation*}

where $\Delta \phi (f) = -\int _0^f [x_{{\rm ssDNA}}(f^{\prime })-x_{{\rm G4}}(f^{\prime })] df^{\prime }$ is the conformational energy change between unfolded ssDNA and the folded telomeric G4.

Due to complex stacking and hydrogen bond formation between the bases, the force response of ssDNA cannot be ideally described by the commonly used worm-like-chain or the freely-joint-chain polymer models. Up to date, the ssDNA force-extension curve *x*_ssDNA_(*f*) is much better described by a phenomenological force–extension relation proposed by Cocco *et al.* (39) (SI: ‘Force-extension curve for ssDNA’). Folded G4 can be approximated as a rigid body with a size of *l*_0_ ∼1.7 nm estimated from NMR structure. Its projected length along the force direction is given by: $x_{{\rm G4}}(f) = l_0 \coth ({f l_0}/{k_{\rm B}T})- {k_{\rm B}T}/{f}$. Based on these force-extension curves and Δ*G*(*f*) data, Δ*G*_0_ of unfolding short- and long-lived state are calculated to be: 3.6 ± 0.6 *k*_B_*T* and 5.9 ± 0.4 *k*_B_*T* (2.1 ± 0.4 kcal mol^−1^ and 3.5 ± 0.2 kcal mol^−1^, average ± standard error), respectively.

## DISCUSSION

In summary, this work has studied the equilibrium folding and unfolding transitions of a single telomeric G4 in physiological potassium concentration using magnetic tweezers. Several important results have been obtained, including identifying three folded structures with distinct life times and mechanical stability. Our observation of a short-lived and a long-lived state for G4 formed in a time scale of several minutes is consistent with previous smFRET studies carried out at similar K^+^ concentrations that reported two folded G4 populations in the absence of force, which were indicted by different levels of FRET efficiency (22,27). In particular, the ultra long-lived state with a high mechanical stability has not been reported in previous experiments. Besides, the force dependent free energy cost of unfolding the telomeric G4 for both the short- and long-lived forms, Δ*G*(*f*), is obtained based on direct equilibrium measurements.

Previous studies have reported a wide range of Δ*G*_0_ in 3.4−14.8 kcal mol^−1^ (23). The value determined from this study is close to the lower boundary of this range. While the causes of such big variation of Δ*G*_0_ from different experiments have remained unclear, we reason that it may be related to the polymorphism of the telomeric G4. Different forms of telomeric G4 may be kinetically separated, which may lead to different G4 population distributions in different experiments depending on the exact folding protocol. This is consistent with a recent study reporting that the population distribution of multiple telomeric G4 structures folded by a slow thermal annealing procedure is distinct from that obtained by quick quench folding (42). It is possible that at a longer time scale, other structures such as ultra long-lived state observed in our study may accumulate, leading to a higher free energy cost for unfolding.

Our data have suggested that folding to the long-lived state is through the short-lived state as an intermediate, which agrees with the conclusion made in a previous smFRET study at a low KCl concentration (2 mM) (22). The presence of an intermediate state between ssDNA and G4 has also been suggested in several previous biochemical, single-molecule and simulation studies, which has been proposed to be a partially folded structure involving 3-folded strands and one peeled strand (38,43,44). The existence of a short-lived intermediate state is in general consistent with these earlier works.

## SUPPLEMENTARY DATA

Supplementary Data are available at NAR Online including [1–5].

SUPPLEMENTARY DATA
