# Supplementary material for: Dynamics and stability of polymorphic human telomeric G-quadruplex under tension
Source: Nucleic Acids Res. 2014 Jul 10;42(13):8789–95. doi: 10.1093/nar/gku581 (PMC4117794; doi:10.1093/nar/gku581)
Supplement: SUPPLEMENTARY DATA [file supp_42_13_8789__index.html]

Dynamics and stability of polymorphic human telomeric G-quadruplex under tension — SUPPLEMENTARY DATA 

# Dynamics and stability of polymorphic human telomeric G-quadruplex under tension

## SUPPLEMENTARY DATA

**Files in this Data Supplement:**

- Supplementary Data
