# Supplementary material for: Dynamics and stability of polymorphic human telomeric G-quadruplex under tension
Source: Nucleic Acids Res. 2014 Jul 10;42(13):8789–95. doi: 10.1093/nar/gku581 (PMC4117794; doi:10.1093/nar/gku581)
Supplement: SUPPLEMENTARY DATA [file supp_gku581_nar-01269-f-2014-File009.pdf]

Supplemental Materials for "Dynamics and stability of polymorphic human telomeric G-quadruplex under tension"

DNA oligonucleotides

G4 oligo:
5'-phosphate-
CTTGTGCACAGACTCGTTGGGTTAGGGTTAGGGTTAGGGTTTGCAGCCAGGTCAGTAGCGAC

Flank1 and Flank2:
5'- CGAGTCTGTGCACAAGGTGC
5'-phosphate-CTACTGACCTGGCTGC

Oligos for PCR:
594l: 5'- CAGTCCCACGTGCATGGCAGGAGGGCGAATG
594r: 5'-Biotin- CGAAGCAGATCCCACGCAACCAGCTTACGG
1417l : 5'- ATCA CCAAGTGCATGGTGCTTGAACCCGCCTATG
1417r : 5'- thiol-CGACTGAGCACGCAACCAGCTTACGG

The 62 nt ssDNA containing the telomeric G4 sequence (blue) was first annealed with flank 1 and flank 2, and then ligated with the two ds DNA handles prepared by PCR reactions. The product construct is shown in Fig. S1.

Data processing

The unfolding force and dwell time were analyzed using an in-house written Matlab program. The unfolding steps during constant loading rate stretching were detected based on abrupt extension change in a 0.1 s sliding time window. This method is similar to that developed by Cui et al. (1). The detailed implementation was published in our previous publication (2). The transition steps at constant forces were detected using a step fitting package "stepfit1" (3), which is optimized for detecting fluctuation between states under low constant forces.

Kinetic models

(i). Sequential model (see illustration in Fig. S7A, left panel)

The probability evolution equations for ssDNA, short-lived and long-lived states are

$$\frac{dP_{ssDNA}}{dt} = -k_{12}P_{ssDNA} + k_{21}P_{short}$$

$$\frac{dP_{short}}{dt} = k_{12}P_{ssDNA} - (k_{21} + k_{23})P_{short} + k_{32}P_{long}$$

$$\frac{dP_{long}}{dt} = k_{23}P_{short} - k_{32}P_{long}$$

(ii). Parallel model (see illustration in Fig. S7A, right panel)

The probability evolution equations for ssDNA, short-lived and long-lived states are

$$\frac{dP_{ssDNA}}{dt} = -k_{12}P_{ssDNA} + k_{21}P_{short} - k_{13}P_{ssDNA} + k_{31}P_{long}$$

$$\frac{dP_{short}}{dt} = k_{12}P_{ssDNA} - k_{21}P_{short}$$

$$\frac{dP_{long}}{dt} = k_{13}P_{ssDNA} - k_{31}P_{long}$$

These sets of differential equations in terms of P_{ssDNA}, P_{short}, P_{Long} were solved using the Runge-Kutta method by the Matlab function ODE45 using the kinetic parameters listed in Tables S1 and S2.

Table S1. Kinetic parameters estimated from dwell time analysis (sequential model)

	k_{12} (s^{-1})	k_{21} (s^{-1})	k_{32} (s^{-1})	k_{21}/k_{23}	k_{23} (s^{-1})
5pN	0.063	0.33	0.022	1.25	0.27
6pN	0.019	0.48	0.041	0.87	0.55
7pN	0.010	0.31	0.060	0.86	0.36

$k_{12} = k_{u\to f}, k_{21} = k_{f\to u,short}, k_{32} = k_{f\to u,long}, \frac{k_{21}}{k_{23}} = \frac{N_{short}}{N_{long}}$

TableS2. Kinetic parameters estimated from dwell time analysis (parallel model)

	$k_{u\to f}$ (s^{-1})	k_{21} (s^{-1})	k_{31} (s^{-1})	k_{12}/k_{13}	k_{12} (s^{-1})	k_{13} (s^{-1})
5pN	0.063	0.33	0.022	1.25	0.035	0.028
6pN	0.019	0.48	0.041	0.87	0.0089	0.010
7pN	0.010	0.31	0.060	0.86	0.0046	0.0053

$k_{12} + k_{13} = k_{u\to f}, k_{21} = k_{f\to u,short}, k_{31} = k_{f\to u,long}, \frac{k_{12}}{k_{13}} = \frac{N_{short}}{N_{long}}$

Force-extension curve for ssDNA

The force-extension curves for ssDNA was described by the following phenomenological relation proposed by Cocco *et* al.

$$x_{ssDNA} = h\left(\frac{a_1 \ln\frac{f}{f_1}}{1 + a_3 e^{-\frac{f}{f_2}}} - a_2 - \frac{f}{f_3}\right)$$

, with the following empirical parameters $h = 0.34$ nm,$a_1 = 0.21$, $a_2 = 0.34$, $f_1 = 0.0037$pN, $f_2 = 2.9$ pN and $f_1 = 8000$ pN (4). The salt dependence is described by $a_3 = 2.1\ln([\mathrm{K}^+]/0.0025)/\ln(0.15/0.0025)$, where $[\mathrm{K}^+]$is the salt concentration expressed in the units of Molar. With these parameters, the model has been shown able to fit the ssDNA force-extension curves over a wide force range (1-100 pN) and a wide salt concentration (> 50 mM KCl) (4). Based on this ssDNA force-

extension curve, the extension change of 21 nt ssDNA folding into G4 at 17 pN is ~ 8 nm, which is consistent with the step size observed in experiments (Fig. S2).

Table S3. Force dependent free-energy cost for unfolding short- and long-lived G4

	$\Delta G_{ssDNA,short}$ (k_BT)	$\Delta G_{ssDNA,long}$ (k_BT)	$\Delta\Phi$ (k_BT)	$\Delta G^0{}_{ssDNA,short}$	$\Delta G^0{}_{ssDNA,long}$
5pN	-0.2	2.3	-3	2.8	5.3
6pN	-0.9	1.7	-4.3	3.4	6.0
7pN	-1.3	0.5	-5.7	4.4	6.2

From the values of $\Delta G^0{}_{ssDNA,short}$ and $\Delta G^0{}_{ssDNA,long}$ evaluated at the three force, their averages and standard errors are determined to be $\Delta G^0{}_{ssDNA,short} = 3.6 \pm 0.6\, k_B T$ and $\Delta G^0{}_{ssDNA,long} = 5.9 \pm 0.4\, k_B T$, respectively.

SUPPLEMENTARY FIGURES

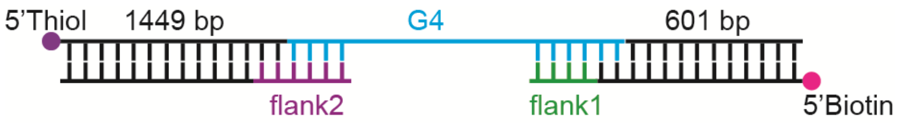

Figure S1. Telomeric G4 sequence (blue) sandwithced between with two dsDNA handles.

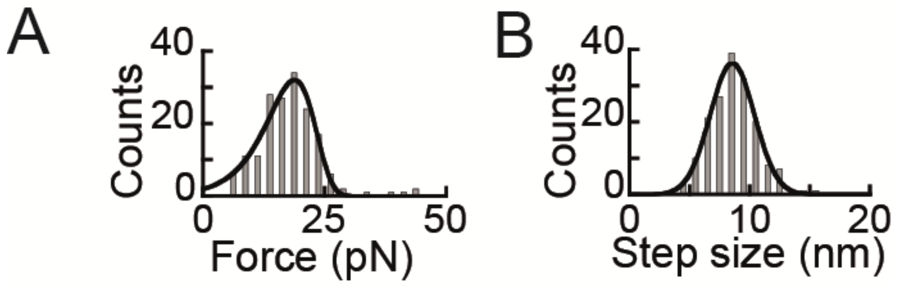

Figure S2. Unfolding force and step size. (A) Unfolding force histogram obtained at 2 pN/s loading rate (171 events collected from 7 molecules). Solid line is obtained by fitting the data with the Bell's model (Eq. 2 in the main text) with $x_u = 0.82 \pm 0.05$ nm and $k_u{}^0 = 0.009 \pm 0.002$ s^{-1} (average ± standard error). (B) Unfolding step-size distributions obtained at 2 pN/s, with a peak value of 8 ± 2 nm by Gaussian fitting (Black curve). The step sizes were determined by a noise-beating step detection algorithm detailed in methods.

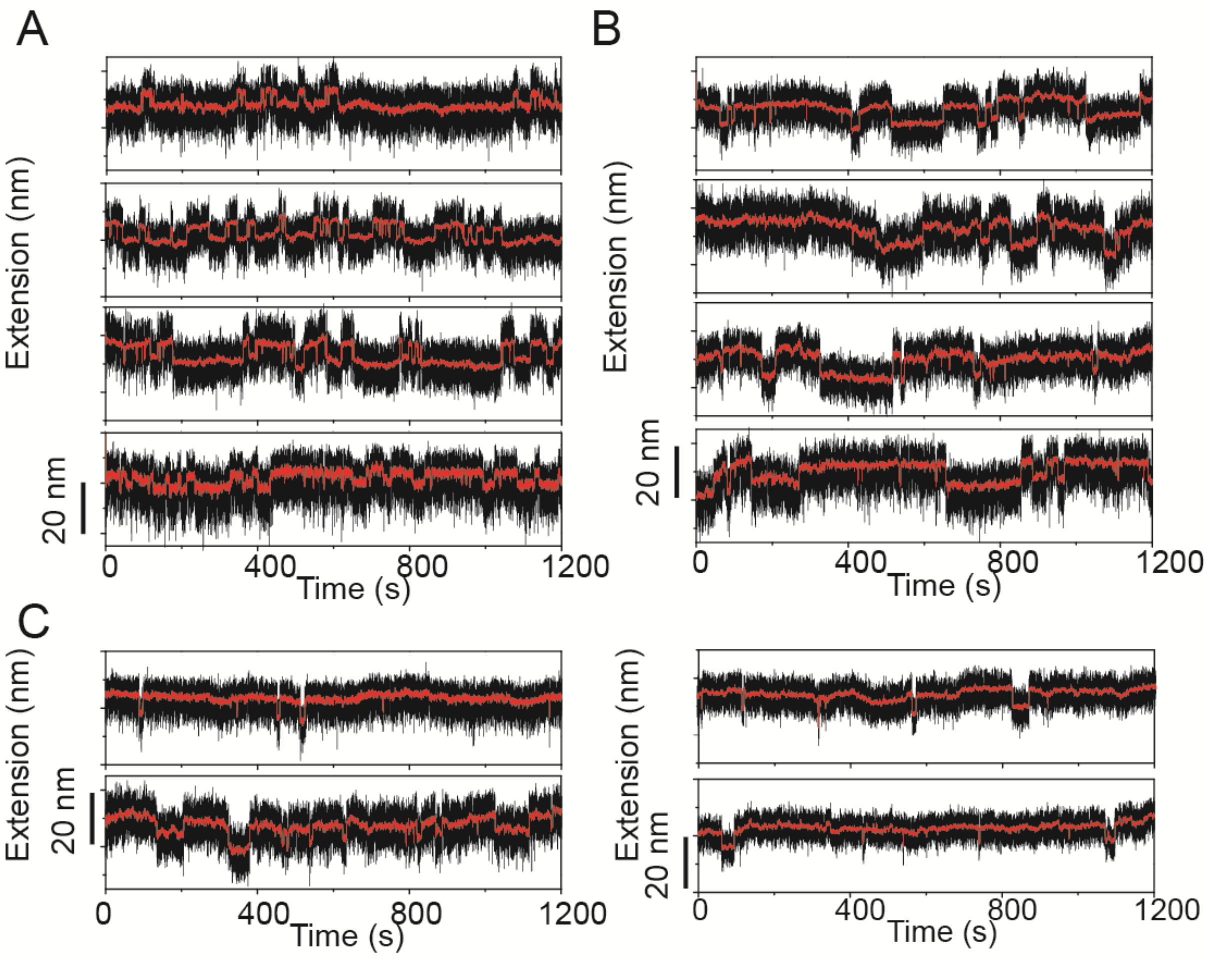

Figure S3. G4 folding and unfolding dynamics under constant forces.

Time trajectories (black dots) of the extension of a single G4 tether recorded at constant forces: 5 pN (A), 6 pN (B), and 7 pN (C). The red line shows smoothed time trace by adjacent average of data using a 0.5-sec sliding time window. For each force, 4 different DNA tethers were plotted.

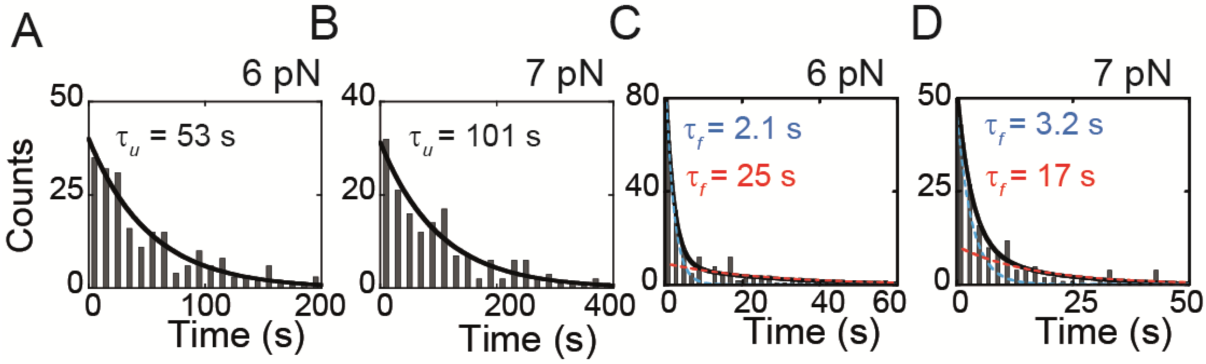

Figure S4. Dwell times of DNA extension states under different forces. Histogram of the dwell time of the longer extension state (i.e., unfolded G4) recorded at 6 pN (A) and 7 pN (B). Data were fitted by a

single-exponential decay function (black curve) with time constant of 53 ± 5 s and 101 ± 11 s respectively. Histogram of the dwell time of the shorter extension state (i.e., unfolded G4) at 6 pN (C) and 7 pN (D). Data were fitted by a double-exponential decay function with two time constant 2.1 ± 0.2 s, 25.0 ± 6.3 s for 6 pN and 3.2 ± 0.5 s, 16.6 ± 4.9 s for 7pN (7pN: 177 events from 10 molecules. 6pN: 219 events from 9 molecules).

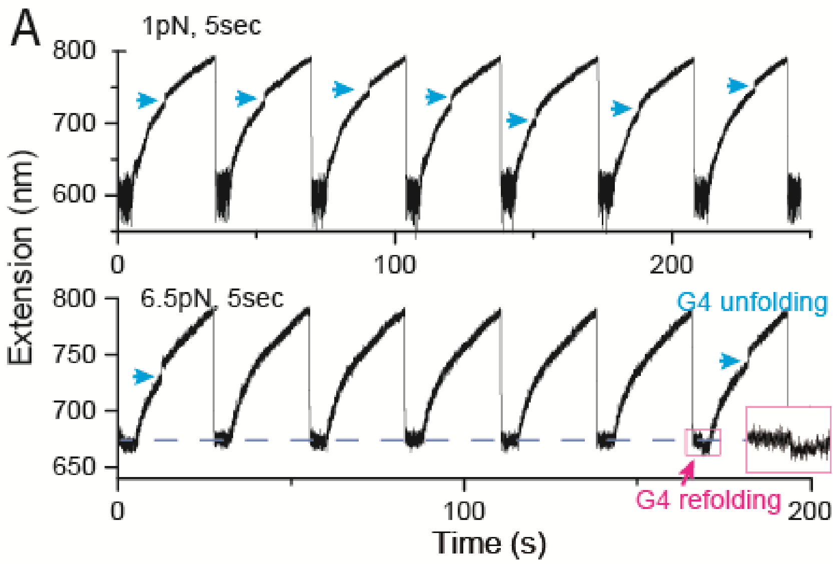

Figure S5. Typical stretching cycles for measuring the refolding probability. Same molecule was hold at 1 pN (upper panel) and 6.5 pN (lower panel) for 5 s and then was stretched at constant loading rate of 2 pN/s. In upper panel, all seven cycles showed G4 unfolding (cyan arrows) extension jump. In contrast, in lower panel, only two unfolding events were detected in total seven stretching cycles.

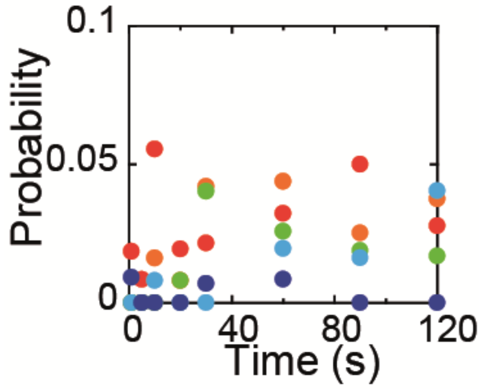

Figure S6. Refolding probability of ultra-long-lived G4. The refolding probability up to 120 s calculated from the unfolding events with unfolding force larger than 32 pN. Based on the unfolding force distribution shown in Fig.1C, 99.7% of the unfolding forces for long-lived state are within 0-32 pN (average ± 3 standard deviations). Data from different forces where refolding occurred were represented by different colors (red, 1pN; orange, 3 pN; green, 5 pN; cyan, 6 pN; blue, 7 pN). Less than 2% events (66 events over 4417 stretching cycle) have unfolding force of higher than 32 pN.

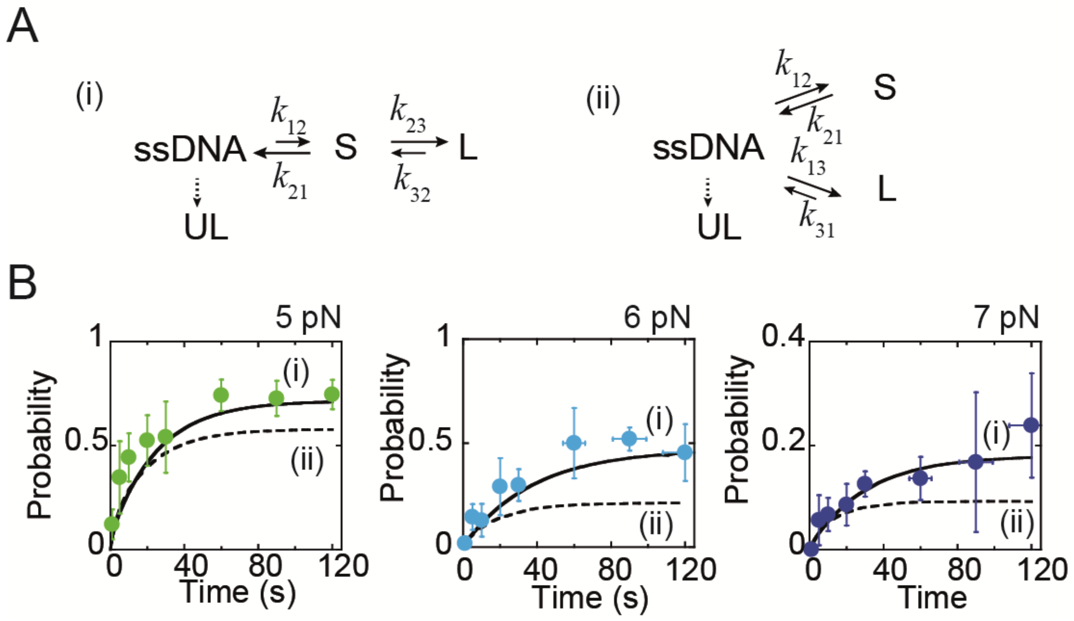

Figure S7. Refolding probability agree with sequential model. (A) Sequential model (i) and parallel model (ii).(B).Comparing the refolding probability data and consecutive model (Black solid line) and parallel model (Black dash line). The theoretical curves were calculated using the folding and unfolding rates determined in dwell time analysis.

1. Cui, Y., Petrushenko, Z.M. and Rybenkov, V.V. (2008) MukB acts as a macromolecular clamp in DNA condensation. *Nature structural & molecular biology*, **15**, 411-418.
2. Le, S., Chen, H., Cong, P., Lin, J., Droge, P. and Yan, J. (2013) Mechanosensing of DNA bending in a single specific protein-DNA complex. *Scientific reports*, **3**, 3508.
3. Aggarwal, T., Materassi, D., Davison, R., Hays, T. and Salapaka, M. (2012) Detection of Steps in Single Molecule Data. *Cellular and molecular bioengineering*, **5**, 14-31.
4. Cocco, S., Yan, J., Leger, J.F., Chatenay, D. and Marko, J.F. (2004) Overstretching and force-driven strand separation of double-helix DNA. *Physical review. E, Statistical, nonlinear, and soft matter physics*, **70**, 011910.
5. Lee, J.Y., Okumus, B., Kim, D.S. and Ha, T. (2005) Extreme conformational diversity in human telomeric DNA. *Proceedings of the National Academy of Sciences of the United States of America*, **102**, 18938-18943.
